# pp32 (ANP32A) Expression Inhibits Pancreatic Cancer Cell Growth and Induces Gemcitabine Resistance by Disrupting HuR Binding to mRNAs

**DOI:** 10.1371/journal.pone.0015455

**Published:** 2010-11-29

**Authors:** Timothy K. Williams, Christina L. Costantino, Nikolai A. Bildzukewicz, Nathan G. Richards, David W. Rittenhouse, Lisa Einstein, Joseph A. Cozzitorto, Judith C. Keen, Abhijit Dasgupta, Myriam Gorospe, Gregory E. Gonye, Charles J. Yeo, Agnieszka K. Witkiewicz, Jonathan R. Brody

**Affiliations:** 1 Department of Surgery, Jefferson Pancreas, Biliary and Related Cancer Center, Thomas Jefferson University, Philadelphia, Pennsylvania, United States of America; 2 Robert Wood Johnson Medical School, University of Medicine and Dentistry of New Jersey, Camden, New Jersey, United States of America; 3 Department of Pathology, Thomas Jefferson University, Philadelphia, Pennsylvania, United States of America; 4 Kimmel Cancer Center, Thomas Jefferson University, Philadelphia, Pennsylvania, United States of America; 5 Laboratory of Cellular and Molecular Biology, National Institute on Aging-Intramural Research Program, National Institutes of Health, Baltimore, Maryland, United States of America; University of Medicine and Dentistry of New Jersey, United States of America

## Abstract

The expression of protein phosphatase 32 (PP32, ANP32A) is low in poorly differentiated pancreatic cancers and is linked to the levels of HuR (ELAV1), a predictive marker for gemcitabine response. In pancreatic cancer cells, exogenous overexpression of pp32 inhibited cell growth, supporting its long-recognized role as a tumor suppressor in pancreatic cancer. In chemotherapeutic sensitivity screening assays, cells overexpressing pp32 were selectively resistant to the nucleoside analogs gemcitabine and cytarabine (ARA-C), but were sensitized to 5-fluorouracil; conversely, silencing pp32 in pancreatic cancer cells enhanced gemcitabine sensitivity. The cytoplasmic levels of pp32 increased after cancer cells are treated with certain stressors, including gemcitabine. pp32 overexpression reduced the association of HuR with the mRNA encoding the gemcitabine-metabolizing enzyme deoxycytidine kinase (dCK), causing a significant reduction in dCK protein levels. Similarly, ectopic pp32 expression caused a reduction in HuR binding of mRNAs encoding tumor-promoting proteins (e.g., VEGF and HuR), while silencing pp32 dramatically enhanced the binding of these mRNA targets. Low pp32 nuclear expression correlated with high-grade tumors and the presence of lymph node metastasis, as compared to patients' tumors with high nuclear pp32 expression. Although pp32 expression levels did not enhance the predictive power of cytoplasmic HuR status, nuclear pp32 levels and cytoplasmic HuR levels associated significantly in patient samples. Thus, we provide novel evidence that the tumor suppressor function of pp32 can be attributed to its ability to disrupt HuR binding to target mRNAs encoding key proteins for cancer cell survival and drug efficacy.

## Introduction

Pancreatic adenocarcinoma (PDA) is an aggressive malignancy with a poor prognosis, even following surgical resection [1], [2]. While 5-fluorouracil (5-FU) and gemcitabine (GEM) with or without radiation therapy constitute standard treatment in the adjuvant setting, they provide little improvement in long-term survival [3], [4], [5]. Therefore, a better understanding of acquired and *de novo* chemotherapeutic resistance mechanisms is necessary for us to enhance current treatment strategies. Although much has been learned about the molecular changes involved in the process of pancreatic tumorigenesis, there has been little success in our understanding of why pancreatic cancer cells are resistant to chemotherapy [6], [7].

pp32 (ANP32A) has a unique pattern of expression in many human cancers [8], [9], [10], [11]. pp32 functions as a tumor suppressor protein [12], as demonstrated by its ability to inhibit *k-ras*-mediated malignant transformation [13], [14]. We previously showed that pp32 expression correlates with the differentiation status of PDA, with normal expression levels detected in well-differentiated tumors but reduced-to-absent expression levels in poorly differentiated tumors [14]. These findings are significant because poorly differentiated forms of PDA are both common and aggressive, yet little is understood about the specific molecular characteristics of this form of PDA [15]. In a previous study, introduction of pp32 into a poorly differentiated pancreatic cell line caused cell cycle arrest and inhibited cell growth [14].

pp32 has been shown to be a binding partner of multiple important proteins [14], [16], [17], [18], [19]. Previous work demonstrated that pp32 is involved in: 1) stabilization of certain mRNAs bearing AU-rich elements (AREs) in the 5′ and 3′ untranslated regions (UTRs) via the interaction of pp32 with the RNA-binding protein HuR (ELAVL1) [18]; 2) the modification of histone acetylation through its role in the inhibitor of acetyl transferase complex (termed INHAT) [17]; and 3) the modulation of the cell cycle through its interaction with the phosphorylated form of Rb [18], [19], [20], [21].

Recently, we also discovered that a binding partner of pp32, HuR [18], [22], is central to GEM efficacy against pancreatic cancer cells [23]. We demonstrated that HuR can associate with deoxycytidine kinase (dCK) mRNA and thus regulate dCK protein expression [23]. This association is enhanced when pancreatic cancer cells are exposed to GEM. Upon GEM exposure, dCK levels increase to metabolize GEM (a nucleoside analog) from a prodrug into its active metabolites. Accordingly, patients treated with GEM whose resected tumors expressed elevated cytoplasmic HuR levels had a >7-fold increase in survival compared to patients with resected tumors expressing low cytoplasmic HuR [23]. This previous work provides the framework to explore HuR and related proteins (pp32) in the context of chemotherapeutic efficacy [24].

The exact role of pp32 as a tumor suppressor gene and in its role in HuR's post-transcriptional regulation of target mRNAs is largely unknown. Previously, pp32 co-immunoprecipitated with HuR in cell culture models and it was shown that pp32's RNA recognition motifs were critical for this interaction [18]. Further, different investigators have claimed that pp32 is strictly nuclear or cytoplasmic. Brennan *et al.* first described pp32 as a protein that can shuttle between the nucleus and the cytoplasm along with HuR [18]. Based on this work, we sought to explore functional links between pp32 and HuR in regard to pancreatic cancer cell survival (i.e., cancer cell growth and GEM efficacy).

## Methods

### Establishment of isogenic pp32-overexpressing and control cell lines

MiaPaCa2 cells were transfected using Lipofectamine (Invitrogen, Carlsbad, CA). Full-length pp32 cDNA was subcloned into the plasmid pc3.1 Zeo (Invitrogen), which possesses a Zeocin™ resistance gene for selection as previously described [14], [23].

For each sample, 5 uL of the VERIFY Antigen Standard Origene overexpression lysate (1 ug/1uL) were placed with 5 uL of 2x SDS Sample Buffer (OriGene Rockville, Maryland). Overexpression of pp32, HuR, or empty vector were driven by a pCMV6-Entry Vector plasmid that added a C-terminal Myc/DDK tag to each gene (OriGene). Samples were prepared and then loaded on a NuPage 10% Bis-Tris Gel and separated at 200 volts for 60 minutes. Proteins were then transferred to a PDVF membrane at 30 volts for 90 minutes. The membrane was blocked for 1 hour. The membranes were probed with primary antibodies (thymidylate synthase, dCK, pp32, HuR, and alpha-tubulin; Santa Cruz Biotechnology, Santa Cruz, CA) overnight. The concentrations for primary antibody were as follows: HuR 1∶1000, dCK 1∶500, TS and alpha tubulin 1∶200. Probed antibodies were then washed with TBST solution and secondary antibody was applied with Santa Cruz goat anti-mouse IgG-HRP antibody at a concentration of 1∶10,000. Membranes were then washed and developed using the Immobilon Western Chemiluminescent HRP Substrate detection system (Millipore, Billerica, MA).

#### Transient transfection of pp32 expression vector and siRNA for Ribonucleoprotein immunoprecipitation binding (RNP-IP) assays

Transient transfection was performed as described above. siRNA knockdown was performed by using a pp32 designed small interfering siRNA (Dharmacon, Thermoscientific) with the use of oligofectamine (Invitrogen) as previously described [9], [23]. In brief, pancreatic cancer cell lines PL5 and MiaPaca2 cells were plated at 60% confluence and transfected in Oligofectamine and Optimem (Invitrogen) using pp32 siRNA and a negative control scramble sequence (Dharmacon). Cells were collected after 48 hours for immunoblot, sensitivity assays, and RNP-IP assays.

### Isolation of RNA and genomic DNA detection of plasmids

To confirm the overexpression and reduction of pp32 mRNA in cell lines, semi-quantitative RT-PCR was performed. MiaPaCa2 pp32-transfected (Mia.pp32) and empty vector (Mia.EV) cells were trypsinized and collected as previously described [25] and our generated do novo using the previously generated and purchased parental pancreatic cancer cell line (ATCC, Manassas, VA). Genomic DNA was isolated from Mia.pp32 and Mia.EV cell lines and plasmid integration was confirmed by performing PCR with a forward primer specific for the T7 sequence of the plasmid and a reverse primer specific for pp32: FWD 5′-TAATACGACTCACTATAGGG-3′, REV 5′-CAGGTTCTCGTTTTCGCTTC-3′.

Total RNA was isolated using RNeasy RNA isolation kit (Qiagen) and then treated with Turbo-DNA*free* (Ambion, Austin, TX) to eliminate trace amounts of gDNA [25].

#### Ribonucleoprotein immunoprecipitation (RNP-IP) and real-time quantitative PCR (qPCR)

Cells were plated at 65% confluency and treated as indicated. Immunoprecipitation was performed using either anti-HuR or anti-IgG control antibodies as previously described (MBL International, Woburn, MA) [23], [26]. RT-PCR was then performed, after mass normalization of RNA samples, to generate cDNA. Optical Density of cDNA was measured and RT-quantitative PCR (qPCR) was performed on an ABI 7500 instrument; 75 ng of cDNA template was used per reaction to determine the relative abundance of dCK, VEGF, and HuR mRNAs; samples were normalized to GAPDH mRNA levels.

### SDS-PAGE/Western Blotting

Mia.pp32 and Mia.EV cells were trypsinized and whole-cell lysates were obtained using RIPA lysis buffer. Protein quantitation was performed using a Bradford assay (BioRad, Hercules, CA). Sample concentrations were equalized using RIPA. Samples were then mixed 1∶1 with 2X Laemmli buffer and separated using a 10% Bis-Tris polyacrylimide gel in 1x MOPS running buffer and proteins were transferred and blotted with indicated antibodies as previously described above [13].

### Immunofluorescence

Mia.pp32 and Mia.EV cells were plated onto chamber slides and treated with the indicated drugs. After treatment, cells were washed in PBS, incubated with the indicated antibody and processed as previously described [23]. Cell nuclei were stained with DAPI and chamber slides were mounted for analysis with a Zeiss LSM-510 Confocal Laser Microscope.

### Cytoplasmic Extracts

MiaPaCa2 cells were plated at 60% confluence. Six h after treatment with 1 µM gemcitabine (Eli Lilly) or no treatment, cytoplasmic extracts were prepared as described [23], [26], and immunoblot analysis performed [23] using primary antibodies that recognized HuR (3A2, 1∶1000, Santa Cruz), hnRNP or pp32 (1∶500) [13].

### Growth assay

Using the same transfection protocol outlined above, MiaPaCa2 parental cells were transfected with equal amounts of pp32-encoding and empty vector pcDNA in T-75 flasks. Media was changed and Zeocin™ selection was performed as described above. At the end of the two-week period, the medium was aspirated and flasks were stained with crystal violet solution for 20 minutes, followed by thorough washes or cells were counted as described in the figure legend.

### Drug Sensitivity Assays

Sensitivity assays were performed using PicoGreen™ (Invitrogen), a fluorescent dye that selectively binds double-stranded DNA. The intensity of the fluorescent signal correlates with the number of viable cells. In brief, 2000 cells were plated per well of a 96-well plate and treated 24 h later [23]. Chemotherapeutic agents were purchased from Sigma unless mentioned otherwise.

### RNA binding assays

For ribonucleoprotein immunoprecipitation (RNP-IP) analysis, MiaPaca2 cells were plated at a 65% confluency, treated 24 h later with 1 µM gemcitabine for 3 h and IP performed using either anti-HuR or IgG control antibodies as described [23], [26]. After RNA isolation, dCK mRNA levels were measured by PCR analysis using primers TCTCTGAATGGCAAGCTCAA and CTATGCAGGAGCCAGCTTTC [23].

### Immunohistochemistry and patient samples

Formalin-fixed, paraffin-embedded blocks were processed as described [23] using heat antigen retrieval and avidin-biotin complex detection. Immunostaining was performed on 37 resected PDA specimens from the Thomas Jefferson University pathology archives after the Thomas Jefferson University Institutional Review Board (IRB). We adhered to all ethical considerations herein. All patient samples used were under the Thomas Jefferson University Institutional Review Board (IRB) approved protocol. Thus this study was performed with 100% patient consent. No new cell lines generated directly from human tissue were used for this study. Consent was written. IRB approval title is “Collection, Banking, and Evaluation of tissues, blood, pancreatic juice and bile from patients with pancreatic and related carcinomas undergoing surgical resection.” In accordance with the US Department of Health and Human services (IRB approved). The majority of patients received GEM alone, or in combination with Xeloda or radiation therapy. Antibodies recognizing pp32 [14] or HuR [23] were used previously [23]. Cellular localization (nuclear versus cytoplasmic) and staining intensity (strong versus weak) were scored. Based on the percentage of stained cells (>50% versus 5–50%) the expression was scored as diffuse or focal, respectively. Survival curves were generated using GraphPad Prism (Version 4.0) and p values calculated using a log-rank (Mantel-Cox) test.

## Results

### Characterization of pp32 overexpressing cell lines

**Figure 1:**
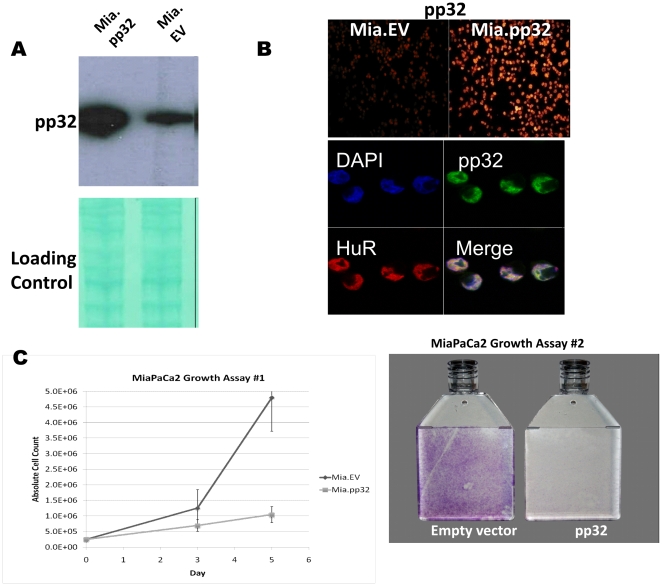
Characterization of pp32-overexpressing cancer cells. (A) Immunoblot analysis of protein lysates from Mia.pp32 cells and controls. Mia.pp32 cells express increased pp32 levels than Mia.EV cells. (B) Immunofluorescence with Mia.pp32 and Mia.EV cells (*top*). Immunofluorescence was also performed with labeling of HuR, pp32, and DAPI under a higher magnification (*bottom*). Cells were then analyzed using laser confocal microscopy. (C) Mia.pp32 cells have significantly reduced growth potential relative to Mia.EV cells. (*Left*) Cells were equally plated and collected on days 3 and 5 and counted. Five-fold fewer Mia.pp32 cells were counted at 5 day compared to Mia.EV cells. (*Right*) MiaPaCa2 cells were transfected with equal amounts of pp32 and empty vector pcDNA 3.1(Zeo). The flasks were treated similarly over a two-week period and subsequently stained with crystal violet to quantitate the number of viable cells (see methods). Each flask is representative of 3 flasks.

Plasmid integration into MiaPaCa2 cells was confirmed by PCR amplification of genomic DNA (data not shown). Figure 1 depicts confirmation of pp32 protein overexpression in the Mia.pp32 cell line relative to Mia.EV. Equal protein loading was confirmed by staining the membrane using Fast Green (Figure 1A). The strong nuclear presence of pp32 in Mia.pp32 cells was detected by immunofluorescence (Figure 1B). Periodic immunoblot analysis was performed to validate continued overexpression of pp32 protein in the Mia.pp32 cells (Figure 1).

#### Pancreatic cancer cells have significantly reduced growth potential compared to control cells

Numerous attempts to generate Hs766T and PL5 cells overexpressing pp32 were unsuccessful, while the empty vector plasmid generated colonies routinely (unpublished data, see Methods)[14]. Similar results were described in previous studies [8], [14].

Mia.pp32 cells routinely required less frequent passaging than Mia.EV cells. Growth assays (Figure 1C, *left*) performed as described (see Methods) revealed that by day 5, there were 5-fold fewer Mia.pp32 cells than Mia.EV cells. Note the typical logartihmic growth of the Mia.EV compared to the blunted, linear growth rate of Mia.pp32. We did not observe significant cell death in either cell line in the sub-confluent state, supporting the conclusion that reduced cell growth, rather than apoptosis, accounted for the dramatic difference in cell counts, as previously described [14].

We transfected the pp32 and empty vector plasmids into equal amounts of parental MiaPaCa2 cells. A dramatic reduction in growth in the MiaPaCa2 cells transfected with pp32 was detected compared to the cells transfected with empty vector. We noted markedly decreased staining in the pp32-transfected flask (Figure 1C, *right*), demonstrating the decreased growth potential of these cells compared to the control. Together, these experiments ruled out the possibility that pp32 reduced cell proliferation due to ‘position-effect variegation’ resulting from the random integration of a gene into an undesirable region in the genome.

### Drug sensitivity assays revealed Mia.pp32 cells to be resistant to nucleoside analogs

**Figure 2:**
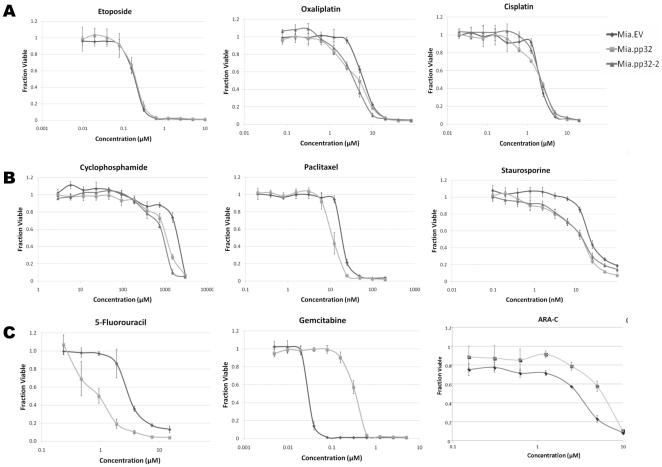
Cell survival assays of Mia.pp32 and Mia.EV cells treated with various chemotherapeutics (**Table 1**). Survival of Mia.pp32 and Mia.EV lines was measured by the PicoGreen assay after 5–7 days of incubation with the indicated drug doses. (A) Drugs that cause no pp32-dependent sensitivity; (B), drugs showing modest differences in sensitivity; (C) drugs for which pp32 conferred enhanced resistance. Graphs represent single experiments (S.E.M.); each experiment is representative of >three individual experiments. Mia.pp32 lines are indicated as ▴▪ and the empty vector control cells are indicated as ♦.

Once stably transfected Mia.pp32 and Mia.EV cell lines were established, cells were treated with various chemotherapeutic agents from different drug classes (Table 1). For most drugs such as etoposide, cisplatin, oxaliplatin (Figure 2A), cyclophosphamide and paclitaxel (Figure 2B, see Table 1 for drug class descriptions) only negligible changes in chemosensitivity were seen between Mia.pp32 and Mia.EV cells (Table 1 and Figure 2A and B). An additional sub-line of pp32 transfected cells (Mia.pp32-2) was included as an experimental control, and differences were found between all pp32 overexpressing cell lines and the empty vector control cells, thus ruling out an artifact of cloning (Figure 2). Both Mia.pp32 lines and Mia.EV proliferated at the same rate, as indicated by negligible differences observed in cell surivival percentages between the cell lines at extreme low doses and concentration of each drug tested (Figures 2A-C).

**Table 1:** Class of drugs used in the drug sensitivity assays performed against Mia.pp32 and Mia. EV cell lines with respective IC50s of each drug identified.

Drugs	Class/Mechanism of Action	Mia.pp32 IC50 Concentration	Mia.EV IC50 Concentration
Etoposide	Mitosis inhibitor; topoisomerase II inhibitor	200 nM	200 nM
Oxaliplatin	Alkylating agent; DNA cross-linker	4.5 µM	6.5 µM
Cisplatin	Alkylating agent; DNA cross-linker	2 µM	2 µM
Cyclophosphamide	Alkylating agent; DNA cross-linker	1 mM	2 mM
Paclitaxel	Mitosis inhibitor; microtubule stabilizer	11 nM	19 nM
Vinblastine	Mitosis inhibitor; microtubule inhibition	300 pM	300 pM
Staurosporine	Protein kinase inhibitor	15 nM	24 nM
5-Fluorouracil	Antimetabolite; pyrimidine analog	1 µM	3 µM
Gemcitabine	Antimetabolite; pyrimidine analog	350 nM	30 nM
ARA-C	Antimetabolite	9 µM	5 µM

There was a modest increase in sensitivity of Mia.pp32 lines to the protein kinase C inhibitor staurosporine (STS) compared to Mia.EV (Figure 2B, *right*). Mia.pp32 cells were two-fold more sensitive to 5-FU compared to Mia.EV cells (Figure 2C, *left*). However, the most dramatic change was noted with drugs from the same class that utilize dCK for cellular metabolism: GEM and cytarabine (ARA-C) (Table 1 and Figure 2C, *center and right*). Mia.pp32 cells displayed a ten-fold resistance to GEM compared to Mia.EV, and a 2-fold resistance to ARA-C (Table 1 and representative data, Figure 2C, *center*).

### siRNA knockdown of endogenous pp32 expression sensitizes cells to gemcitabine

**Figure 3:**
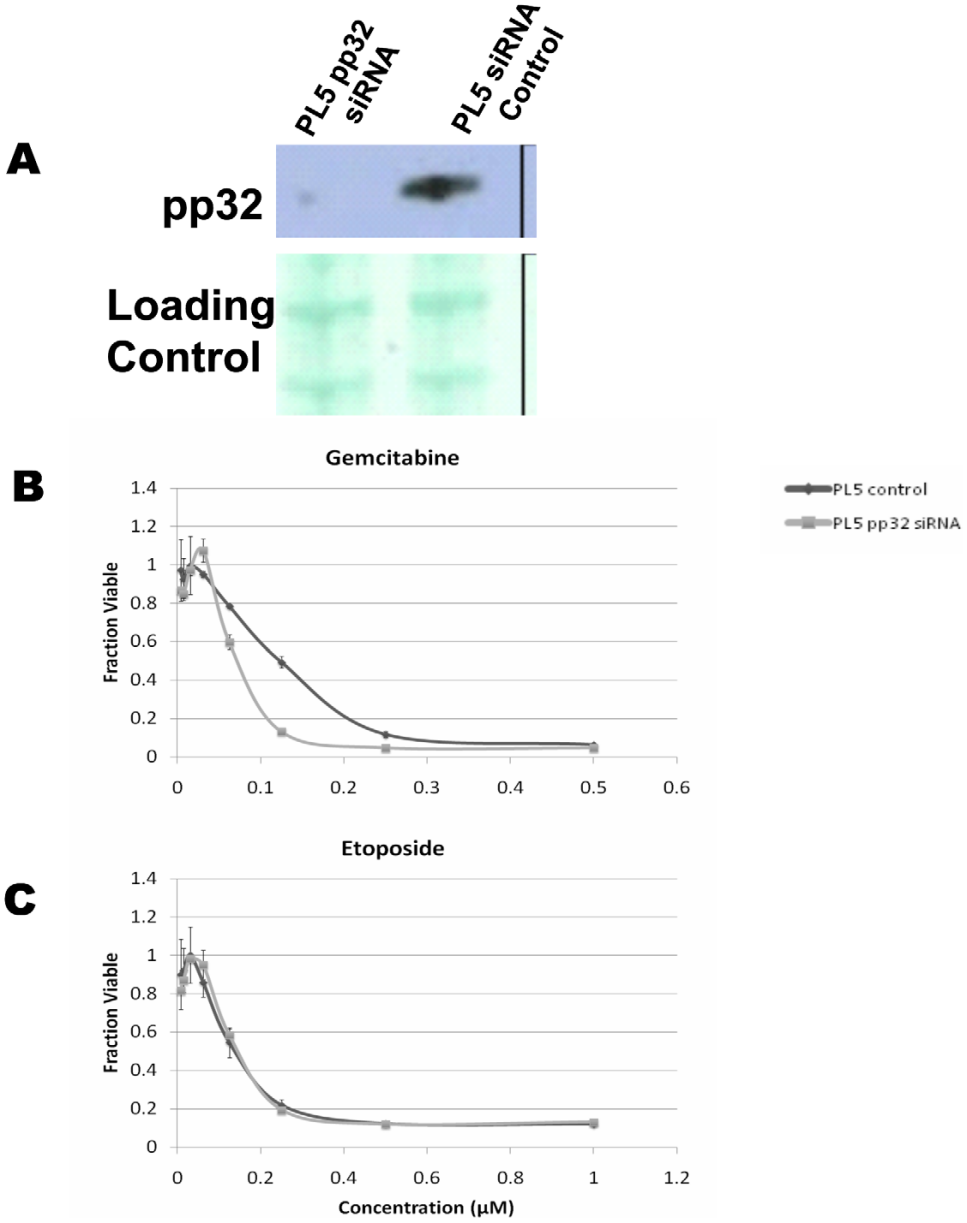
siRNA knock down of pp32 increased sensitivity to GEM. (A) Immunoblot analysis of pp32 abundance in lysates from PL5 cells 48 h after transfection. In cells transfected as explained in (A), the sensitivity to GEM (B) or etoposide (C) was tested by PicoGreen cell survival assay.

The pancreatic cancer cell line PL5, with abundant pp32 expression, was transiently transfected using either pp32 siRNA or a control scrambled sequence. Knockdown of pp32 expression (Figure 3A) rendered cells approximately 3 fold more sensitive to GEM compared to control cells (Figure 3B). pp32 knockdown did not affect cell viability following etoposide (a negative control) treatment (Figure 3C). We did not observe any changes in cell growth parameters or cellular phenotype in the pp32 siRNA cells.

### Overexpression and reduction of pp32 disrupts VEGF, HuR, and dCK mRNA transcript binding to HuR and reduces dCK protein expression

**Figure 4:**
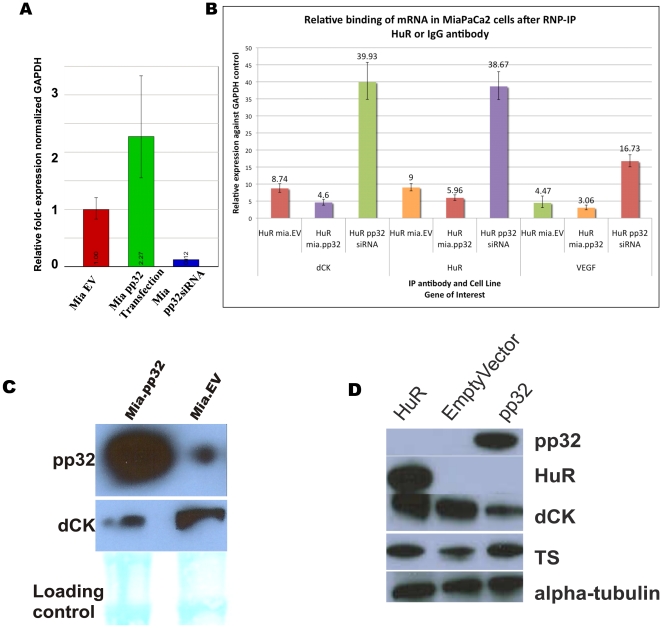
pp32 expression disrupts the association of HuR with dCK, HuR, and VEGF mRNAs. (A) pp32 mRNA levels normalized to GAPDH mRNA levels in empty-vector transfected cells, pp32 siRNA transfected cells, and pp32 plasmid transfected cells. Number indicates fold change of pp32 mRNA expression of labeled generated cell lines compared to empty vector control cells. (B) HuR binding to VEGF and dCK mRNAs was detected by RNP-IP analysis in MiaPaCa2 cells transfected with pp32 siRNA, pp32 plasmid, or empty vector control (A). mRNA levels in HuR and IgG IP samples were first normalized to GAPDH mRNA levels in the same IP reactions, and then plotted as relative fold enrichment in VEGF, dCK, and HuR mRNAs in HuR IP vs IgG IP. Data show the mean from 3 independent data points. Two independent experiments were performed in order to confirm the results. Numbers indicate fold changes compared to IgG control. (C) Western blot analysis of pp32 and dCK expression levels in protein lysates from Mia.pp32 and Mia.EV cells. (D) Three lanes represent lysates from HEK293T cells generated that either left to right: overexpress HuR, empty vector, or pp32 tagged with myc/DCK. Western blot analysis included antibodies recognizing pp32, HuR, dCK, alpha-tubulin, and thymidylate synthase (TS) proteins.

Previously we demonstrated that dCK mRNA binds to HuR and thus enhances dCK protein translation [23]. We manipulated pp32 expression levels (Figure 4A) in isogenic cancer cells and then quantitatively assessed the association of known HuR mRNA targets dCK [23], vascular endothelial growth factor (VEGF) [27], and HuR [28] mRNAs with HuR by ribonucleoprotein immunoprecipitation (RNP-IP) assay as described previously [23]. After a brief GEM treatment, the association between HuR and dCK mRNA was detected in MiaPaCa2 cells (Figure 4B). However, a significant reduction in dCK, VEGF, and HuR mRNAs was detected in HuR antibody-immunoprecipitated-RNA from cells overexpressing pp32 (Figure S1 and Figure 4A and B); while in the pp32 siRNA-transfected cells a significant, consistent enhancement (>4-fold) in dCK, VEGF, and HuR mRNAs bound to HuR (Figure 4A and B). Fold changes were determined by comparing HuR antibody-immunoprecipitated-RNAs from transfected cells to empty-vector transfected cells, with normal, endogenous pp32 expression levels. For specificity, we evaluated and did not find any binding of GAPDH and pp32 mRNAs (Figure 4B and data not shown). Figure S1 shows the dramatic effect of stable overexpression of pp32 (Mia.pp32 cells) have on dCK mRNA binding to HuR.

Additionally, we found that dCK protein levels were reduced in Mia.pp32 cells compared to control cells (Figure 4C). Finally, we utilized a different cell culture model to validate these findings. Protein lysates from Human HEK293T cells (see methods) that overexpressed HuR, pp32 and a control vector. Validation of HuR and pp32 overexpression was confirmed by immunoblotting (Figure 4D). As expected, we detected enhanced dCK protein expression in the HuR overexpression lysates [23] when compared to control and decreased dCK protein expression in the pp32 overexpression lysates when compared to control (Figure 4D). Alpha-tubulin and thymidylate synthase were used to show equal protein loading. These data confirm that dCK is upregulated in a setting when HuR is overexpressed and downregulated in a setting when pp32 is overexpressed. Taken together, these data indicate that pp32 can affect both dCK mRNA binding to HuR and dCK protein expression (Figure 4).

### STS and GEM enhanced cytoplasmic pp32 abundance

**Figure 5:**
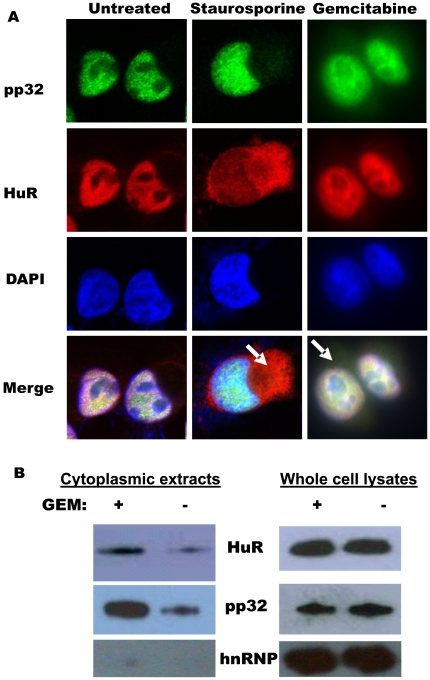
Subcellular localization of pp32 and HuR levels and sensitivity to stressors. (A) Immunofluoresence showing increased HuR and pp32 cytoplasmic expression in cells treated with STS (1 µM for 3 h) and GEM (1 µM for 3 h), as indicated by the white arrows. (B)Immunoblot analysis of HuR and pp32 levels in cytoplasmic and whole-cell lysates prepared from cells that were treated as explained in (Figure 5).

We confirmed previous reports [29] that STS can increase the cytoplasmic levels of both HuR and pp32 in cancer cells (Figure 5A). Similarly, GEM treatment increased pp32 cytoplasmic abundance, but to a lesser extent than HuR (Figure 5A). The increase in pp32 and HuR cytoplasmic levels after GEM treatment was assessed by Western blot analysis (Figure 5B). No change in pp32 and HuR expression was detected in whole-cell lysates from GEM-treated cells (Figure 5B), in agreement with our previous results [23]. Monitoring the levels of hnRNP (C1/C2) confirmed the purity of the cytoplasmic lysates (Figure 5B).

### Nuclear pp32 intensity is a biomarker for poor prognosis in PDA but does not enhance the predictive value of HuR for GEM treatment

**Figure 6:**
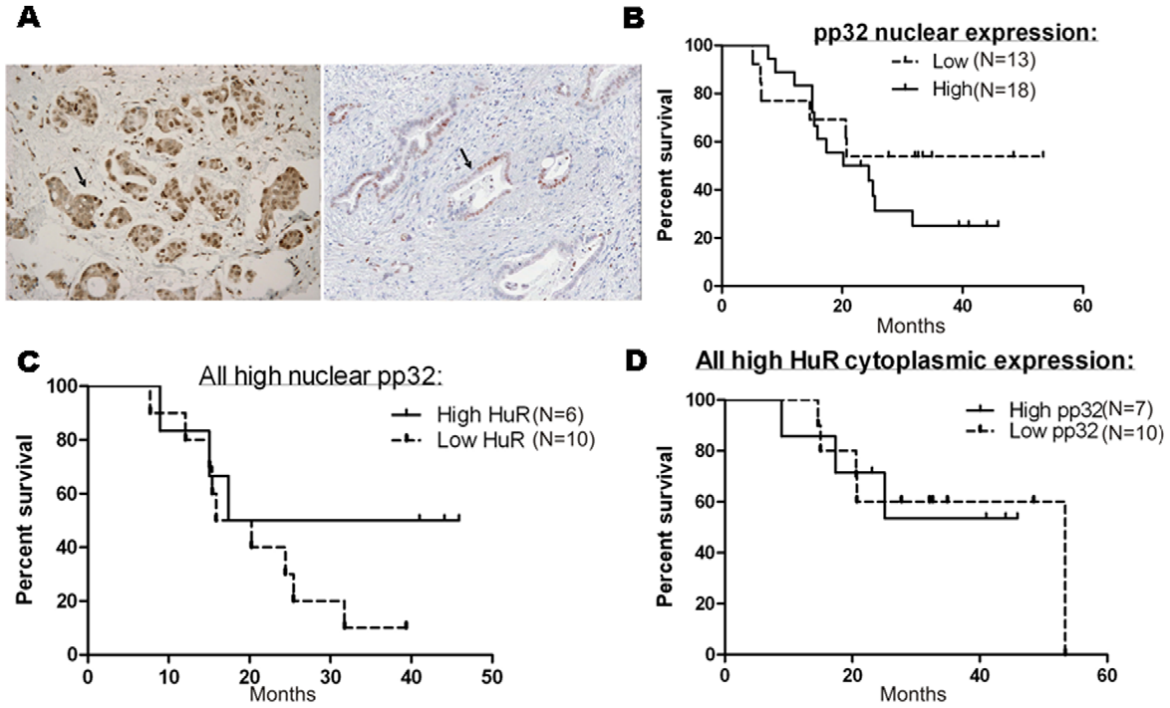
pp32 and HuR expression in clinical samples and patient outcomes. (A) The abundance and subcellular localization of HuR (*left*) and low to absent nuclear pp32 expression (*right*) in samples from pancreatic cancer patients were assessed by immunohistochemistry; magnification, 200x. Samples are representative of the cohort analyzed in B–D. (B) Correlation between pp32 nuclear expression and response to GEM treatment (p = 0.3, log rank test). (C) Correlation between high nuclear pp32-expressing tumor samples stratified into high or low HuR status in regards to GEM response (p = 0.88, log rank test). (D) Correlation between high cytoplasmic HuR-expressing tumor samples stratified into high and low pp32 nuclear expression correlated with GEM response (p = 0.25, log rank test).

We separately detected both strong and weak nuclear and cytoplasmic expression of both HuR and pp32 in PDA specimens [14], [23] (Table 2 and Figure 6A, HuR, *left panel* and pp32, *right panel*). For all patients treated with GEM (n = 31) [23], pp32 nuclear intensity did not correlate significantly with GEM response in regard to overall survival (Figure 6B). pp32 nuclear expression levels in combination with HuR cytoplasmic status (Figures 6C and D) did not enhance the predictive value of HuR alone as a marker for GEM response (p = 0.0009, data now shown [23]). We found a modest association between pp32 and HuR subcellular localization expression levels (Table 3). Table 2 describes the association between low nuclear pp32 levels and more aggressive tumors (higher grade, p = 0.0002, and positive for lymph node metastasis, p = 0.0069, see Table 2). This evidence supports our previous findings, in a separate clinical data set, showing that low pp32 expression correlated with poorly differentiated PDAs [14], [15].

**Table 2:** Association of pp32 nuclear expression with clinicopathologic features.

Clinicopathologic features	pp32 nuclear expression		P value (fisher's exact test)	
	High (n = 19)	Low (n = 18)		
Tumor grade				
1	31%(6)	0%(0)	0.0002†	
2	69%(13)	50%(9)		
3	(0)	50%(9)		
Stage				
T1	5%(1)	6%(1)	Not significant	
T2	32%(6)	17%(3)		
T3	63%(12)	77%(14)		
Lymph node				
No metastases	63%(12)	17%(3)	0.0069	
Metastases	27%(7)	83%(15)		

† indicates the p value between Grades 1 and 3.

**Table 3:** Correlation between pp32 and HuR subcellular localization.

	pp32 nuclear	
HuR cyto	High	Low
High	8	13
Low	11	5

p value = 0.09, Fisher's exact test.

## Discussion

**Figure 7:**
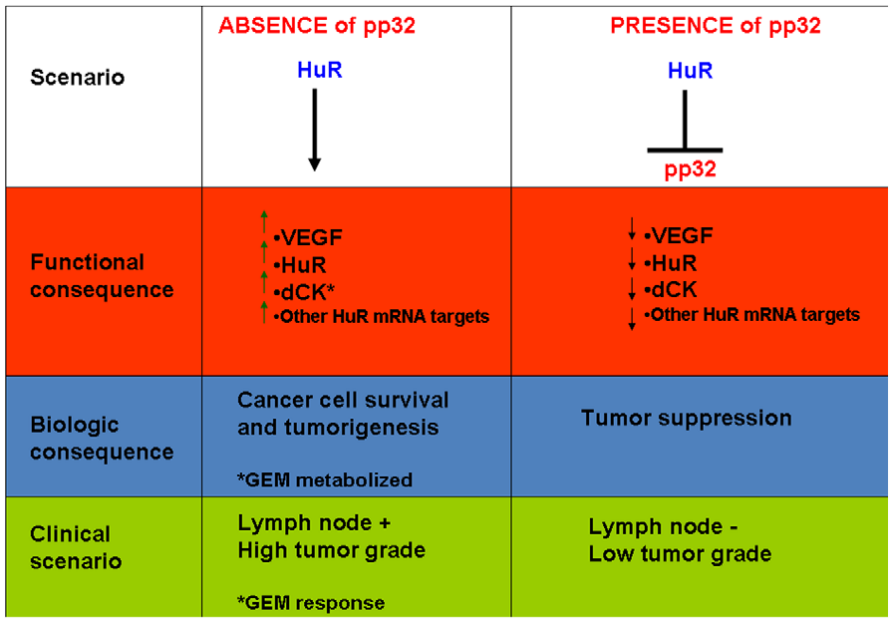
Schematic depiction of the functional, biologic, and clinical consequences of pp32 expression levels on HuR's post-transcriptional regulation of targets including dCK, VEGF, and HuR mRNAs. On the left side shows a scenario where pp32 is reduced or absent (tumorigenesis) and HuR is available to associate and stabilize mRNAs that support cancer cell survival and viability. On the right side is a scenario in which pp32 is present (tumor suppression) and HuR can not bind to mRNAs important for cancer cell survival. Note: GEM is more likely to be metabolized from its prodrug form to its active metabolites by dCK in the scenario on the left.

Numerous researchers have independently characterized pp32 as a tumor suppressor protein in a variety of experimental models [8], [9], [12], [13], [30], [31]. Early studies showed that pp32, through a specific domain comprised of ∼25 amino acids, acted like a tumor suppressor by inhibiting K-ras, a mutant p53, c-jun, E1A, E6, and E7 [12], [13]. We found a strong correlation between both high-grade tumors and lymph node metastasis with weak pp32 nuclear expression (Table 2), supporting our previous findings that pp32 functions as a tumor suppressor protein in PDA [14]. Our results suggest that pp32 expression levels directly disrupt or facilitate HuR's ability to support cancer cell viability and proliferation by disrupting the stabilization of mRNA transcripts encoding proteins necessary for tumor cell survival, such as dCK, VEGF, or HuR (Figure 7). We postulate that the presence of pp32 can disrupt HuR's role in supporting tumorigenesis and cancer cell survival, while the absence of pp32 facilitates tumorigenesis. Our work supports and expands over a decade of research that has proven that pp32 acts like a tumor suppressor gene in multiple models and tumor systems [8], [9], [11], [12], [13], [30], [31], [32] (Figure 7).

Modulation of pp32 expression through overexpression or silencing altered the sensitivity of cancer cells to the nucleoside analogs GEM and ARA-C (Figures 2C and 3). Further, enhanced or reduced pp32 expression levels directly altered the interaction of HuR with dCK mRNA (Figures 4) and significantly lowered dCK protein expression (Figures 4C and D). These data indicate that pp32 plays a role in HuR's post-transcriptional regulation of dCK. We verified earlier reports that cytoplasmic pp32 levels can increase in the presence of specific stressors [22], [33]. Perhaps different stressors transport different pp32 gene family members in conjunction with HuR. For example, Fries B *et al.* demonstrated that *APRIL* and not pp32 acts as a ligand and can aid HuR in its transcriptional regulation of CD83 [33]. Members of the pp32 protein family (e.g., APRIL, pp32r1, pp32) likely provide additional regulatory mechanisms for HuR and its target mRNAs [31].

Although our data provide strong evidence that pp32 modulates HuR's function, our clinical data show that, prior to treatment, endogenous pp32 expression and subcellular localization does not alter HuR's predictive value of GEM response (Figure 6). Moreover, while an association was found between pp32 and HuR subcellular localization in tumor specimens (Table 3), it appears that each protein does not completely regulate the subcellular localization of the other *in vivo*. Several possibilities may explain this finding including the concept that the influence of pp32 on HuR's modulation of dCK expression may be transient, and thus important at the time immediately after drug exposure. We also note the paradoxic differences in sensitivity of our cells to two antimetabolites, 5-FU and GEM, underscoring the importance of further investigation of how the pp32-HuR network may respond uniquely to different chemotherapeutic stimuli and specifically to DNA-damaging agents [24], [34], [35]. Further, STS was more effective in the Mia.pp32 cells (Figure 2B, *right*) and can stimulate transport of pp32 to the cytoplasm (Figure 5), indicating that STS-metabolizing and/or sensitizing gene may be regulated by the HuR/pp32 system.

We postulate four possibilities to explain how pp32 may contribute to HuR's regulation of target mRNAs, and thus GEM efficacy and tumor suppression. First, pp32 may interact with HuR in the nucleus and disrupt target mRNA binding to HuR. Second, pp32 binding of a HuR-mRNA complex may block the ability of the complex to be transported to the cytoplasm. Third, pp32 may retain the HuR-mRNA complex in cytoplasmic foci [22] inhibiting dCK mRNA from proper translation, however this is less likely since we do not detect punctate distribution of pp32 (Figure 5). Fourth, it is possible that disruption of pp32's interaction with HuR (through low pp32 expression levels, subcellular localization, and/or phosphorylation [36]) would allow a HuR-mRNA complex to arrive at the polysomes for enhanced dCK translation. Ongoing studies are aimed at elucidating the exact mechanism(s) whereby pp32 affects HuR's regulation of different target mRNAs in cancer cells. Finally, we can not rule out the possible contribution of other pp32 tumor suppressor functions [12], [17], [19], [29] that may help explain our observations.

In conclusion, we demonstrate that pp32 and HuR have a complex molecular interplay that has clinical relevance with regard to chemotherapeutic efficacy (i.e., GEM response) and cancer cell survival. Subtle changes in pp32 expression levels may potently inhibit multiple core signaling pathways involved in tumorigenesis (Figure 7). By targeting pp32's molecular interaction with HuR, we may be able to achieve improved clinical outcomes for this devastating disease. Future studies will uncover the specificity and the extent in which pp32 can influence all HuR mRNA targets.

## Supporting Information

Figure S1 **RNP IP assay to measure the association of dCK mRNA with HuR in Mia.pp32 cells.** RNA extracted from the RNP IP assays were run as a control (the two right lanes next to the dH_2_0 lane). Equal amounts of RNA converted to labeled cDNA (100 ng each) were amplified via PCR with dCK-specific primers. Labeled ctrl cDNA was RNA converted to cDNA from MiaPaCa2 parental cells and was used as control for the PCR amplification (the right two lanes).
(TIF)
