# Supplementary figures and images for: pp32 (ANP32A) Expression Inhibits Pancreatic Cancer Cell Growth and Induces Gemcitabine Resistance by Disrupting HuR Binding to mRNAs
Source: PLoS One. 2010 Nov 29;5(11):e15455. doi: 10.1371/journal.pone.0015455 (PMC2994932; doi:10.1371/journal.pone.0015455)

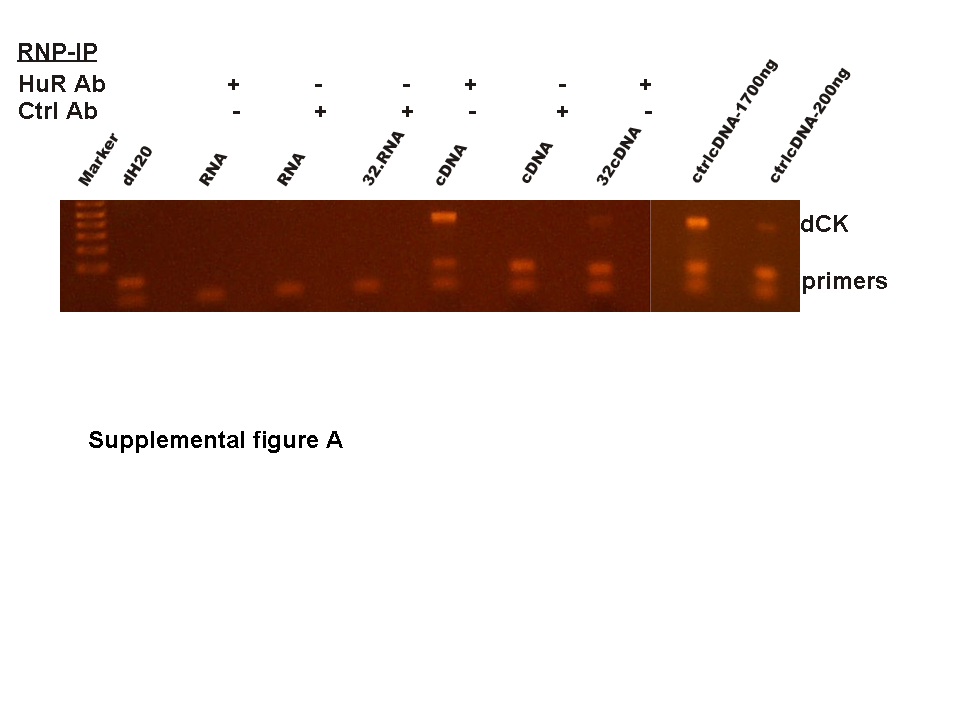

Supplement: Figure S1 — RNP IP assay to measure the association of dCK mRNA with HuR in Mia.pp32 cells. RNA extracted from the RNP IP assays were run as a control (the two right lanes next to the dH20 lane). Equal amounts of RNA converted to labeled cDNA (100 ng each) were amplified via PCR with dCK-specific primers. Labeled ctrl cDNA was RNA converted to cDNA from MiaPaCa2 parental cells and was used as control for the PCR amplification (the right two lanes). (TIF) [file pone.0015455.s001.tif]
